# CRISPRpas: programmable regulation of alternative polyadenylation by dCas9

**DOI:** 10.1093/nar/gkab519

**Published:** 2021-07-09

**Authors:** Jihae Shin, Qingbao Ding, Luyang Wang, Yange Cui, Erdene Baljinnyam, Aysegul Guvenek, Bin Tian

**Affiliations:** Department of Microbiology, Biochemistry and Molecular Genetics, Rutgers New Jersey Medical School, Newark, NJ 07103, USA; Department of Microbiology, Biochemistry and Molecular Genetics, Rutgers New Jersey Medical School, Newark, NJ 07103, USA; Program in Gene Expression and Regulation, the Wistar Institute, Philadelphia, PA 19104, USA; Program in Gene Expression and Regulation, the Wistar Institute, Philadelphia, PA 19104, USA; Program in Gene Expression and Regulation, the Wistar Institute, Philadelphia, PA 19104, USA; Department of Microbiology, Biochemistry and Molecular Genetics, Rutgers New Jersey Medical School, Newark, NJ 07103, USA; Department of Microbiology, Biochemistry and Molecular Genetics, Rutgers New Jersey Medical School, Newark, NJ 07103, USA; Rutgers School of Graduate Studies, Newark, NJ 07103, USA; Department of Microbiology, Biochemistry and Molecular Genetics, Rutgers New Jersey Medical School, Newark, NJ 07103, USA; Program in Gene Expression and Regulation, the Wistar Institute, Philadelphia, PA 19104, USA; Center for Systems and Computational Biology, the Wistar Institute, Philadelphia, PA 19104, USA

## Abstract

Most human protein-coding genes produce alternative polyadenylation (APA) isoforms that differ in 3′ UTR size or, when coupled with splicing, have variable coding sequences. APA is an important layer of gene expression program critical for defining cell identity. Here, by using a catalytically dead Cas9 and coupling its target site with polyadenylation site (PAS), we develop a method, named CRISPRpas, to alter APA isoform abundance. CRISPRpas functions by enhancing proximal PAS usage, whose efficiency is influenced by several factors, including targeting strand of DNA, distance between PAS and target sequence and strength of the PAS. For intronic polyadenylation (IPA), splicing features, such as strengths of 5′ splice site and 3′ splice site, also affect CRISPRpas efficiency. We show modulation of APA of multiple endogenous genes, including IPA of *PCF11*, a master regulator of APA and gene expression. In sum, CRISPRpas offers a programmable tool for APA regulation that impacts gene expression.

## INTRODUCTION

Cleavage and polyadenylation (CPA) is essential for 3′ end maturation of almost all eukaryotic mRNAs (1). The site for CPA, commonly referred to as polyA site or PAS, is defined by surrounding sequence motifs (2,3). While the upstream A[A/U]UAAA hexamer or other close variants are the most prominent motif of PAS (4,5), other upstream sequences, such as UGUA and U-rich motifs, as well as downstream sequences, such as U-rich and GU-rich motifs, additionally enhance PAS usage, often in a combinatorial manner (6–9). Mutations changing the PAS strength have been reported in a growing number of human diseases, including thalassemia and systemic lupus erythematosus (10,11). Moreover, recent studies have found that single nucleotide polymorphisms (SNPs) near the PAS can lead to changes in PAS usage and gene expression (12,13).

Most mammalian genes have multiple PASs, resulting in expression of alternative polyadenylation (APA) isoforms containing different coding sequences and/or 3′ untranslated regions (3′UTRs) (14,15). Most APA events take place in 3′UTRs, named 3′UTR APA events, which change the 3′UTR length, thereby regulating 3′UTR motifs involved in aspects of mRNA metabolism, including stability, translation, and subcellular localization (16). In addition, a sizable fraction of genes contain APA sites in introns, whose usage leads to transcripts encoding distinct proteins (17,18). Similar to 3′UTR APA, intronic polyadenylation (IPA) can play important roles in gene expression in development and disease (17–21). While the biological importance of APA is increasingly appreciated, experimental strategies to modulate PAS usage are still limited.

The CRISPR/Cas9 system has emerged as a powerful tool for genome editing (22). The catalytically dead Cas9 (dCas9) has also been used for transcriptional inhibition or activation thanks to its efficiency in interaction with its target DNA (23–25). Cas9-mediated editing of PAS was employed in several recent studies to examine specific APA isoforms (19,26–28). However, genome editing permanently changes the DNA sequence, making it difficult to examine short-term effects. A programmable APA at the RNA processing step would therefore be desirable in certain experimental settings. Here we present a non-genomic editing method, named CRISPRpas, to alter APA. CRISPRpas delivers dCas9 to the downstream region of a target PAS. By blocking the progression of RNA polymerase II (Pol II), dCas9 promotes the usage of upstream PAS. We demonstrate effective APA isoform changes using reporter constructs and with multiple endogenous genes, including *PCF11*, a key global APA and gene expression regulator. We elucidate several features that affect the efficacy of CRISPRpas, including target strand selection, distance from PAS to target site, and PAS strength. When in the context of IPA, we further examine the importance of features influencing splicing kinetics.

## MATERIALS AND METHODS

### Cell culture and transfection

Human HEK293T and HeLa Tet-On cells were cultured in high glucose Dulbecco's modified Eagle's medium with 10% Fetal Bovine Serum (FBS, Gibco) and 1% Penicillin/Streptomycin solution (Sigma). All cells were incubated at 37°C with 5% CO_2_ and routinely checked by EVOS FL Auto Cell Imaging System (Thermo Fisher).

### Molecular cloning of plasmids

Information for plasmid construction is shown in the Supplementary Table S1.

### gRNA design

gRNA sequences were either designed using CRISPOR (29) which calculates gRNA specificity score as described in a previous study (30) or were based on previous publications. Oilgos were annealed and inserted into the pGR9 plasmid (containing a Cas9 gRNA scaffold sequence) digested with BbsI. Oligos used for gRNA cloning are listed in Supplementary Table S2. Chemically synthesized 2′-O-Methyl phosphorothioate-modified (first and last three residues) gRNAs were obtained from Genscript (Piscataway, NJ, USA). Synthetic gRNA sequences are shown in Supplementary Table S3.

### Flow cytometry analysis

Cells transfected with reporter plasmids for 48–72 h were collected by using trypsinization. Green and red fluorescent signals were measured on a BD LSRFortessa X-20 machine (excitation: 488 and 561 nm; emission: 520/30 and 585/15 nm, respectively). Untransfected cells were used to determine background level. Signals were analyzed using BD digital software (DIVA). Cells negative in both red and green signals were filtered. Log_2_(Red) and log_2_(Red/Green) were calculated for each cell.

### Generation of a stable cell line expressing dCas9

The PiggyBac system was used to generate a stable cell line expressing dCas9. Briefly, HEK293T cells growing on a 12-well plate were transfected with HyPB7 and PiggyBac expression plasmids (System biosciences) using lipofectamine 3000 (Thermo Fisher) with 1 μg of total DNA. Cells were selected with 400 μg/ml hygromycin for 6 days followed by monoclonal selection and expansion. Successful genomic integration was confirmed by microscopy and western blot analysis. One single clone was established and named HEK293T^dCas9^.

### Transfection for reporter assays

A mixture containing 200 ng of reporter construct, 200 ng of dCas9-encoding plasmid, and 100 ng of gRNA-encoding plasmid was transfected into HeLa Tet-On cells seeded in a 24-well plate using Lipofectamine 3000. Culture media was changed the next day with fresh media containing 2 μg/ml doxycycline (Dox). Induction of the tetracycline response element (TRE) promoter was carried out for 2 days. Alternatively, a mixture containing 200 ng of reporter construct and 400 ng of gRNA-encoding plasmid was transfected into HEK293T^dCas9^ cells.

### Transfection of gRNAs for endogenous genes

HEK293T^dCas9^ cells were seeded in a 12-well plate 1 day before transfection. 1 μg of pGR-sgRNA plasmid was transfected using Lipofectamine 3000 according to manufacturer's protocol. Alternatively, 37.5 nM of synthetic sgRNA oligos were used per well. RNA samples were collected after 48 h, and protein samples were collected after 72 h.

### RT-qPCR

Total RNA was collected with TRIzol (Invitrogen). Residual genomic DNA was digested with TURBO DNase (Invitrogen) followed by inactivation of the enzyme. cDNA was synthesized from 2 μg of total RNA using M-MLV reverse transcriptase (Promega) with an oligo(dT)_18-25_ primer. cDNA was mixed with gene-specific primers and then subject to reverse transcriptase-quantitative polymerase chain reaction (RT-qPCR) using Hot Start Taq-based Luna qPCR master mix (NEB). The reaction was run on a Bio-Rad CFX Real Time PCR system. Primers were designed to amplify specific APA isoforms, when needed. Primer sequences are listed in Supplementary Table S4. A Δ*C*_t_-value was calculated for the two primer sets used for isoform analysis. Two-tailed student's *t*-test was used to calculate significance of difference between ΔCt values of primer sets between gRNA and Ctrl gRNA samples.

### Western blot

Protein concentration was determined using the DC Protein Assay (Bio-Rad). A total of 20 μg of protein per sample was resolved using 4%-15% TGX stain-free gels (Bio-Rad), followed by immunoblotting using PCF11 (Proteintech, 23540–1) or GAPDH (CST, 5174) antibodies. Peroxidase AffiniPure donkey anti-rabbit IgG antibody was used as a secondary antibody (Jackson, 711-035-152). Clarity ECL reagent (Bio-Rad) was used to generate chemiluminescent signals, which were captured by the ChemiDoc Touch Imaging System. Signals were analyzed in ImageJ program.

### 3′READS+ sequencing of newly-made and pre-existing RNA

For 4-thiouridine (4sU) labeling and fractionation, cells were cultured with 50 μM of 4sU (Sigma) for 1 h. Total cellular RNA (100 μg) was subject to biotinylation with biotin-HPDP. Labeled RNAs, representing newly made RNAs, were captured by Streptavidin C1 Dynabeads (Thermo Fisher). Unbound flow-through (FT) RNAs were also collected to represent pre-existing RNAs. The 4sU and FT RNAs were subjected to 3′READS+ sequencing, as previously described (31). Briefly, input RNA was captured on oligo(dT)_25_ magnetic beads and fragmented with RNAse III on the beads. Partially digested poly(A)+ RNA fragments were ligated to a 5′ adapter (5′-CCUUGGCACCCGAGAAUUCCANNNN) with T4 RNA ligase 1. The ligated products were incubated with biotin-5′-T_15_-(+TT)_5_, where +T is locked deoxythymidine, and digested by using RNase H. Digested products were ligated to a 3′ adapter with T4 RNA ligase 2. The final ligation products were reverse-transcribed, followed by PCR amplification with index primers for multiplex sequencing. PCR products were size-selected with AMPure XP beads (Beckman) and quality checked with ScreenTape (Agilent). Libraries were sequenced on an Ilumina HiSeq (2 × 150 paired-end reads).

### Stability Score analysis

3′READS+ data were processed and analyzed as previously described (32). Briefly, 5′ adapter and 3′ adapter sequences were first removed. Reads were then mapped to the human genome (hg19) using bowtie2 (v2.2.9) (local mode) (33). Reads with a mapping quality score (MAPQ) <10 were discarded. Reads with ≥2 non-genomic 5′ Ts after alignment were called PAS reads. PASs within 24 nt from each other were clustered as previously described (14). Stability Score of each PAS isoform is log_2_(Ratio) of its reads per million mapped (RPM) value in the FT fraction to that in the 4sU fraction. Stability Scores of all PASs detected in HEK293T cells are provided in Supplementary Table S5.

### QuantSeq sequencing and data analysis

Total cellular RNA was subjected to RNA sequencing by using the QuantSeq FWD kit. Library preparation and sequencing were carried out by Admera Health (South Plainfield, NJ, USA). QuantSeq data were analyzed according to the analysis pipeline from Lexogen. Briefly, raw reads were first trimmed using the BBtools script bbduk (https://sourceforge.net/projects/bbmap/). The remaining sequences were mapped to the human genome (hg19) using STAR-2.7.7a (34). BAM files of two replicates were merged. APA analysis with QuantSeq data was carried out by using the MAAPER program (https://github.com/Vivianstats/MAAPER). For 3′UTR APA analysis, the two PASs in the 3′UTR of the last exon with the greatest changes between two comparing samples were selected. For IPA analysis, one PAS in the 3′UTR of the last exon and one PAS located in an intron with the greatest changes, respectively, between two samples were selected. Relative Expression Difference (RED) was calculated as the difference in log_2_(Ratio) of the abundances of two PAS isoforms between two samples. Significant APA events were those with *P* < 0.05 (Fisher's exact test).

## RESULTS

### CRISPRpas alters PAS usage

**Figure 1.**
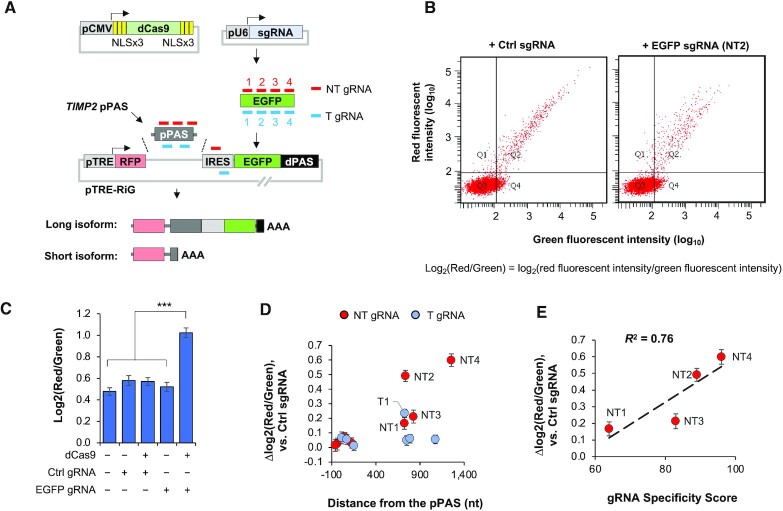
CRISPRpas alters 3′UTR PAS usage in a reporter construct. (**A**) Schematic of a reporter system to test CRISPRpas. mRNA expression of a dCas9 protein tagged with multiple copies of NLS is driven by a CMV promoter. A U6 promoter drives the expression of gRNAs that base-pair with either the non-template (NT) or template (T) strand of target DNA. gRNA target locations are indicated (not drawn to scale). The pTRE-RiG vector expresses a short isoform using the proximal PAS (pPAS) and a long isoform using the distal PAS (dPAS). TRE promoter is used for expression under the control of Doxycycline. Human *TIMP2* proximal PAS was used as pPAS in the vector. dPAS is SV40 early PAS. AAA, poly(A) tail. (**B**) Representative images of flow cytometry data of HeLa Tet-On cells co-transfected with pCMV-dCas9, pTRE-RiG (TIMP2 pPAS) and gRNA-expressing vectors. Each dot is a cell. X and Y axes indicate green and red fluorescent intensity values, respectively. Log2(red/green) is calculated to indicate the relative red versus green fluorescent signals in each cell. (**C**) Comparison of log_2_(red/green) in different samples. ‘+’ and ‘−’ denote presence and absence, respectively. EGFP gRNA NT2 was used. Error bars are standard error of mean. ***, *P* < 0.001 (student's *t*-test). (**D**) Scatter plot showing distance of gRNA target site from the pPAS versus Δlog_2_(red/green) for eight NT and seven T gRNAs as compared to ctrl gRNA. Top five gRNAs based on Δlog_2_(red/green) are indicated on the plot. (**E**) Scatter plot showing gRNA specificity score versus Δlog_2_(red/green) for four NT gRNAs. gRNA Specificity Score is based on the CRISPOR program. *R*^2^ is indicated in the plot.

We hypothesized that a catalytically dead Cas9 (dCas9) that hinders Pol II elongation (25) might promote the usage of proximal PAS (pPAS) versus usage of distal PAS (dPAS). For simplicity, we named this approach CRISPRpas. We first tested the method in a reporter system pTRE-RiG (Table S1), which, upon the treatment of doxycycline, produced two APA isoforms due to the placement of two PASs in the vector (illustrated in Figure 1A). Usage of its pPAS leads to a short isoform encoding RFP only, while usage of dPAS (derived from SV40 early PAS) leads to a long isoform encoding both RFP and EGFP. As such, flow cytometry analysis of red and green fluorescent signals from each cell, calculated as log_2_(Red/Green), can be employed to interrogate the relative expression of the two APA isoforms.

We inserted a 180 nucleotide (nt) sequence containing the pPAS of human *TIMP2* gene into the pTRE-RiG vector (Figure 1A). The pTRE-RiG-TIMP2 plasmid was co-transfected into HeLa Tet-On cells with a plasmid encoding dCas9 with nuclear localization signals (NLSs) and a plasmid encoding a gRNA targeting the EGFP region (NT2, Figure 1A) which was previously shown to be effective in CRISPRi (25). Compared to cells transfected with non-targeting control (Ctrl) gRNA, those with NT2 gRNA showed an increased red to green fluorescent signal ratio [log_2_(red/green), Figure 1B and C], indicating that the NT2 gRNA increased the relative expression level of short APA isoform versus that of long APA isoform. By contrast, expression of Ctrl gRNA or NT2 gRNA alone did not have such an effect (Figure 1C), neither did expression of dCas9 with Ctrl gRNA (Figure 1C). Therefore, APA regulation by CRISPRpas was dCas9-dependent and specific to the gRNA target region.

We next examined several other gRNAs that targeted different loci and/or different strands (Figure 1A). Based on the difference in log_2_(red/green) in cells transfected with target gRNA versus Ctrl gRNA, or Δlog_2_(red/green), we found that none of the template strand (T) gRNAs had any effect on isoform changes, with the exception of T1 (blue dot, Figure 1D). By contrast, four non-template (NT) gRNAs (NT1-4) showed noticeable changes of APA (red dots, Figure 1D). This DNA strand-specific regulation is in line with the fact that CRISPRpas works by blocking Pol II elongation (25). The effectiveness on APA change for the four NT gRNAs generally correlated with their target specificity scores as calculated by CRISPOR (29,30,37) (*R*^2^ = 0.76, Figure 1E).

Interestingly, none of the gRNAs with target sites close to pPAS (within 200 nt) elicited APA changes, regardless of their target strand (Figure 1D), indicating the importance of the distance between gRNA target site and PAS. Note that NT1 and NT3 gRNAs, whose target sites are close to one another (770 versus 872 nt), showed similar APA regulation, despite their difference in target specificity scores (Figure 1E), suggesting that distance to PAS may be more critical than Specificity Score for CRISPRpas. Together, these results indicate that delivery of dCas9 to the NT strand of DNA can alter the usage of upstream PAS and the distance between target site and PAS is important for its effectiveness.

**Figure 2.**
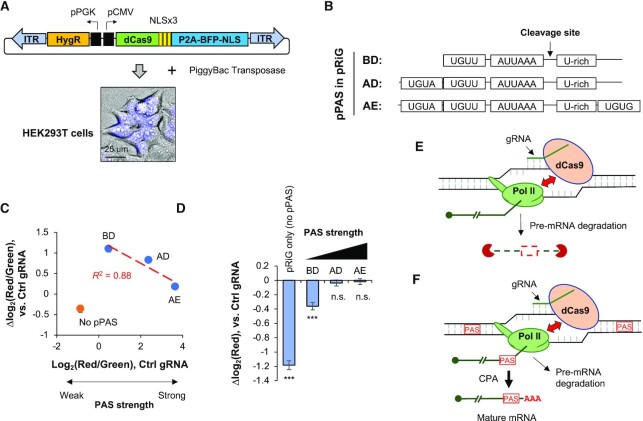
PAS strength affects CRISPRpas efficacy. (**A**) Schematic of creating a stable HEK293T cell line expressing dCas9 using the PiggyBac transposase system. Top, the construct that expresses dCas9-NLSx3-P2A-BFP-NLS and hygromycin-resistant gene. P2A encodes a self-cleaving peptide, and BFP is blue fluorescent protein. ITR, inverted terminal repeat sequence used for genomic integration. Bottom, nuclear BFP signals detected by fluorescence microscopy in HEK293T^dCas9^ cells. A scale bar is shown. (**B**) Schematic of key motifs surrounding three PAS variants derived from the intronic PAS of human *CSTF3*, namely, BD, AD and AE. These PASs were used as pPAS in pRiG. (**C**) Scatter plot showing log_2_(red/green) using Ctrl gRNAs (indicating PAS strength) versus Δlog_2_(red/green) (indicating isoform change) by EGFP NT2 gRNA as compared to Ctrl gRNA in HEK293T^dCas9^. pPAS variants are indicated in (B). Linear regression line is for BD, AD and AE only. pRiG without pPAS is also indicated. (**D**) Bar graph showing Δlog_2_(red), which indicates red fluorescence in cells transfected with EGFP NT2 gRNA as compared to Ctrl gRNA. Significance of each bar compared to 0 is indicated (student's *t*-test). ***, *P* < 0.001; n.s., not significant. (**E**) A model for dCas9-mediated pre-mRNA degradation. When Pol II is stalled by dCas9, pre-mRNA is subject to degradation, either when associated with Pol II or after falling off from Pol II. (**F**) A model for dCas9-mediated APA regulation. When a gRNA targets a region between two PASs, pre-mRNA is subject to CPA at the proximal PAS (left one) when Pol II is stalled. It is also possible that some fraction of pre-mRNA is subject to degradation (also indicated). AAA, poly(A) tail.

Encouraged by our initial results, we next established a cell line, named HEK293T^dCas9^, in which the dCas9-coding sequence was inserted into the genome through the PiggyBac transposase system (Figure 2A). Note that the dCas9 protein was tagged with P2A-BFP-NLS, and hence the level of dCas9 could be monitored by nuclear blue fluorescence signals (Figure 2A).

PASs can have different strengths depending upon surrounding motifs (38). To investigate the effect of PAS strength on CRISPRpas, we transfected HEK293T^dCas9^ with a set of pRiG plasmids (using the CMV promoter) containing PASs with variable flanking sequences (BD, AD and AE, Figure 2B). These PASs were derived from the human *CSTF3* gene (35,36) and deletion mutations of surrounding sequences rendered them to have different strengths. As indicated by flow cytometry analysis, the PAS strength is AE > AD > BD (x-axis, Figure 2C). Using the NT2 gRNA to EGFP and Ctrl gRNA (Figure 1A), we found that APA regulation by CRISPRpas, based on Δlog_2_(red/green) (NT2 versus Ctrl), was most effective with pRiG-BD, followed by pRiG-AD and then pRiG-AE (y-axis, Figure 2C), indicating that CRISPRpas works better when the PAS is weak. In fact, a negative correlation could be discerned between PAS strength and the level of APA regulation by CRISPRpas (red line, *R*^2^ = 0.88, Figure 2C). It is worth noting that CRISPRpas did not function when there was no PAS (pRiG empty vector, Figure 2C, orange dot), indicating that APA regulation by CRISPRpas is PAS-dependent.

We next asked whether dCas9 would change the overall expression level of target sequence as it does in CRISPRi (25). To this end, we measured the level of RFP expression as a proxy for gene expression. We found that CRISPRpas with pRiG plasmid without pPAS led to a significant decrease of RFP expression (*P* = 3.8 × 10^−13^, Wilcoxon test, Figure 2D), consistent with the CRISPRi effect. A milder but also significant decrease of RFP expression was observed for pRiG-BD (*P* = 6.5 × 10^−4^, Wilcoxon test, Figure 2D). By contrast, no RFP downregulation could be discerned for pRiG-AD or pRiG-AE (*P* > 0.05, Wilcoxon test, Figure 2D). A plausible explanation is that CRISPRi-based mechanism, where blocking of Pol II elongation by dCas9 leads to pre-mRNA degradation (depicted in Figure 2E), is in competition with CRISPRpas. In other words, if CPA does not take place within a time window created by dCas9-elicited Pol II stalling, pre-mRNA degradation would take place (depicted in Figure 2F). While a weak PAS is more amenable than a strong PAS for regulation by CRISPRpas, the former is also more prone to pre-mRNA degradation. This view is also in line with the importance of distance between PAS and target site (above), which presumably correlates with the length of the time window in CRISPRpas.

### Systematic snalysis of poly(A)+ RNA isoform stability

We next wanted to use CRISPRpas for endogenous genes. Because APA isoforms can have different mRNA stability levels (31,39), APA isoform abundance changes could be attributed to the combined effect of (i) alteration in APA site and (ii) isoform stability difference. To untangle these two, which could help better interpret our CRISPRpas results, we set out to systemically assess mRNA stability differences between APA isoforms in HEK293T cells.

**Figure 3.**
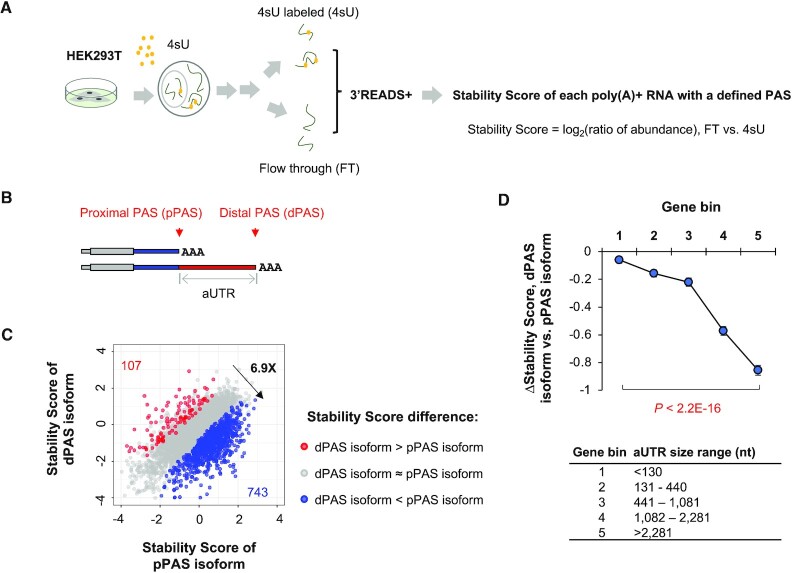
Global analysis of 3′UTR APA isoform stability. (**A**) Schematic showing the experimental procedure. HEK293T was treated with 4sU for 1 h, and total cellular RNA was extracted and subject to fractionation into 4sU-labeled (4sU) and flow-through (FT) fractions. Fractionated RNAs were sequenced by using 3′READS+. Stability Score for each transcript with a defined PAS corresponds to the ratio (log_2_) of reads per million mapped (RPM) in the FT sample to that in the 4sU sample. (**B**) Schematic showing two 3′UTR APA isoforms. They are named proximal PAS (pPAS) and distal PAS (dPAS) isoforms, respectively. The region between the two PASs is named alternative UTR (aUTR). (**C**) Scatter plot showing Stability Score differences between 3′UTR APA isoforms. Two APA isoforms using PASs in the last exon were selected and compared. Genes whose distal PAS (dPAS) isoform were more stable than proximal PAS (pPAS) isoform are shown in red and those with the opposite trend are shown in blue (*P* < 0.05, DEXSeq, *n* = 2). The ratio of these two numbers (6.9×) is also indicated. (**D**) ΔStability Score (dPAS isoform versus pPAS isoform) for genes with different aUTR sizes. Genes were divided into five evenly sized bins based on aUTR size. *P*-value (Wilcoxon test) for significance of difference between bins 1 and bin 5 is indicated. aUTR size range for each bin is shown at bottom.

We first metabolically labeled cellular RNA with 4-thiouridine (4sU) in HEK293T cells for 1 h, and then fractionated RNA into 4sU-labeled and non-labeled (flow-through, or FT) pools (Figure 3A). Both RNA samples were then subjected to 3′ end sequencing by using the 3′ region extraction and deep sequencing (3′READS+ version) method (see ‘Materials and Methods’ section for detail). For each transcript with a defined PAS, we calculated a Stability Score based on the log_2_(ratio) of its abundance in the FT sample, representing pre-existing RNAs, to that in the 4sU-labeled sample, representing newly synthesized RNAs (Figure 3A).

Overall, we detected over 100,000 PASs in HEK293T cells (two replicates). For simplicity, we selected the top two most abundant 3′UTR APA isoforms of each gene (both PASs were in the last exon) for comparison of their Stability Scores (illustrated in Figure 3B). We found that the genes whose short 3′UTR isoform had a higher Stability Score than long 3′UTR isoform (ΔStability Score >0 & *P* < 0.05, DEXSeq) outnumbered those showing the opposite trend (ΔStability Score <0 & *P* < 0.05, DEXSeq) by 6.9-fold (743 versus 107, Figure 3C), indicating a global trend that short 3′UTR isoforms were generally more stable than long 3′UTR isoforms. This result is consistent with our previous finding with mouse NIH3T3 cells (31).

We next divided genes into five bins based on the size difference between selected short and long isoforms, also called alternative UTR (aUTR) size (illustrated in Figure 3B). Based on median ΔStability Score (long isoform versus short isoform) of each gene bin, we found that the longer the aUTR size, the greater the difference between the two isoforms (Figure 3D). For example, genes with an aUTR size >2281 nt (nt, bin 5, top 20%) showed much lower ΔStability Score than genes with an aUTR size <130 nt (bin 1, bottom 20%, *P* < 2.2 × 10^−16^, Wilcoxon test, Figure 3D). Taken together, our global mRNA stability analysis results indicate that long 3′UTR isoforms in general are less stable than short 3′UTR isoforms.

### Regulation of endogenous 3′ UTR APA by CRISPRpas

**Figure 4.**
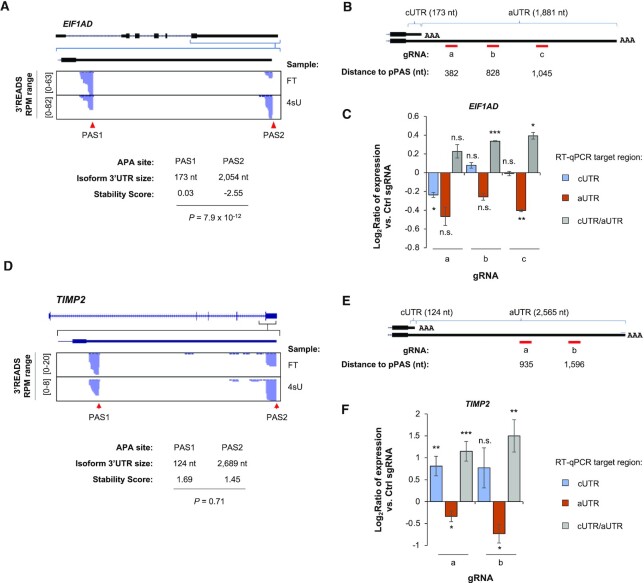
CRISPRpas modulates 3′UTR APA of endogenous genes. (**A**) UCSC tracks showing human *EIF1AD* gene structure (top) and 3′READS+ data for FT and 4sU fractions in the indicated region (bottom). 3′UTR sizes for the isoforms using the two APA sites and their Stability Scores are also shown. *P*-value (DEXSeq, *n* = 2) for Stability Score difference between the two isoforms is indicated. (**B**) Schematic of three gRNAs for *EIF1AD*. Distance to the pPAS is indicated (not drawn to scale). cUTR and aUTR are common UTR and alternative UTR, respectively. Their sizes are indicated. (**C**) RT-qPCR analysis result of relative amounts of cUTR (blue) and aUTR (orange) RNAs in HEK293T^dCas9^ cells transfected with indicated gRNAs (for 48 h). Data are normalized to those of Ctrl gRNA. The ratio of cUTR/aUTR RNA abundances is also indicated (gray). Error bars are standard error of mean (*n* = 2). *P-*value (student's *t*-test) for significance of difference from 0 is indicated. n.s., not significant; *, *P* < 0.05; **, *P* < 0.01; ***, *P* < 0.001. (**D**) As in (A), except that data for *TIMP2* gene are shown. (**E**) As in (B), except that the values are for *TIMP2*. (**F**) As in (C) except that data for two *TIMP2* gRNAs are shown. Error bars are standard error of mean (*n* = 6 and 4 for gRNA-a and gRNA-b, respectively).

We next carried out CRISPRpas for an endogenous gene *EIF1AD* (eukaryotic translation factor 1A domain containing), which expressed two 3′UTR isoforms with a large size difference in HEK293T cells (173 versus 2054 nt, Figure 4A). Based on Stability Scores, the short 3′UTR isoform was substantially more stable than the long 3′UTR isoform (0.03 versus −2.55, *P* = 7.9 × 10^−12^, Figure 4A). We designed three gRNAs targeting the aUTR of *EIF1AD* (gRNA-a, -b and -c, Figure 4B), which were 382, 828 and 1045 nt away from the pPAS, respectively (Figure 4B).

Using RT-qPCR with primer pairs for common UTR (cUTR) and aUTR sequences, respectively, we found that gRNA-b and gRNA-c led to significantly increased cUTR/aUTR expression ratio (Figure 4C), indicating CRISPRpas was in effect. Interestingly, suppression of gene expression, as indicated by the expression level of cUTR sequence compared to GAPDH mRNA, was observed with gRNA-a, the closest one to the pPAS, but not with gRNA-b or gRNA-c (Figure 4C). One likely explanation is that pre-mRNA degradation may have taken place in cells transfected with gRNA-a, but not with gRNA-b or gRNA-c. Thus, this result again underscores the importance of distance between PAS and target site for effective CRISPRpas. On the other hand, no significant increase of cUTR signals was observed with gRNA-b or gRNA-c (Figure 4C), suggesting that stability difference between isoforms does not play a role in our analysis.

We next tested *TIMP2* gene (TIMP metallopeptidase inhibitor 2), which expressed two 3′UTR isoforms also with large size differences in HEK293T cells (124 versus 2,565 nt, Figure 4D). Interestingly, these two isoforms did not show a significant difference in stability (Stability Scores = 1.69 and 1.45, respectively, *P* = 0.71, DEXSeq, Figure 4D). We designed two gRNAs, gRNA-a and gRNA-b, whose target sites were 935 and 1596 nt away from pPAS, respectively (Figure 4E). Using RT-qPCR with primers to cUTR and aUTR, we found that both gRNAs significantly increased cUTR/aUTR ratio (Figure 4F). In both cases, cUTR expression also increased, despite that gRNA-a was statistically significant but gRNA-b was not (Figure 4F). Since the two isoforms are not significantly different in stability, the increase of cUTR should be due primarily to greater usage of pPAS.

We additionally tested CRISPRpas with *CCND1* (cyclin D1) and *CKS1B* (CDC28 protein kinase regulatory subunit 1B), which expressed three and two 3′UTR isoforms in HEK293T cells, respectively (Supplementary Figure S1). gRNA target sites were designed to be far away from the pPAS (1318 and 434 nt, respectively, Supplementary Figure S1A and C). In both cases, the cUTR/aUTR ratio significantly increased (Supplementary Figure S1B and D).

Since gRNAs can also be chemically synthesized, we next compared our plasmid-based gRNAs with synthetic gRNAs (with 5′ and 3′ end 2′-O-Methyl modifications, see ‘Materials and Methods’ section for detail) for CRISPRpas. Using *TIMP2* gRNA-a, we found that synthetic gRNAs worked more effectively (by ∼2-fold, Supplementary Figure S2) than plasmid-based, U6 promoter-driven gRNAs. The effect was already discernable 24 h after transfection, which was not the case for plasmid-based gRNAs (data not shown). While we cannot rule out the possibility that transfection efficiency may also account for the difference to some degree, this result indicates that synthetic gRNAs function rapidly and effectively for CRISPRpas.

### CRISPRpas regulates intronic polyadenylation of a reporter gene

**Figure 5.**
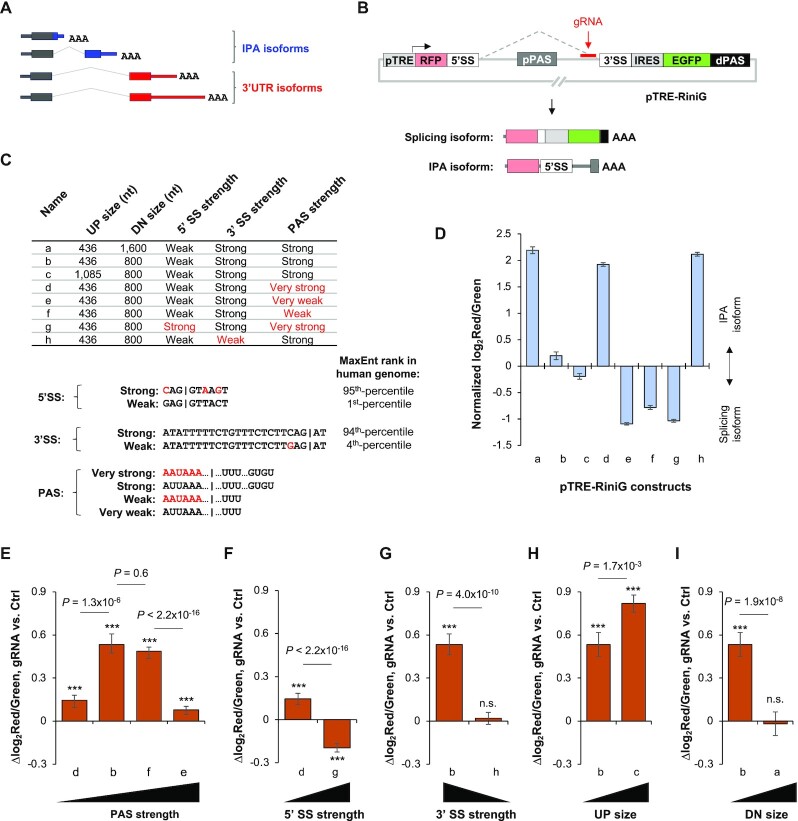
CRISPRpas regulates IPA in a reporter system. (**A**) Schematic of IPA isoforms and 3'UTR isoforms, the latter of which use PASs in the last exon. (**B**) Schematic of pTRE-RiniG reporter construct to examine IPA regulation. The gRNA used is indicated (not to scale). 5′ splicing site (5′SS), 3′ splice site (3′SS) and pPAS were derived from human *CSTF3* gene. Splicing isoform encodes RFP, IRES and EGFP sequences, whereas the IPA isoform encodes RFP and 5′SS. (**C**) A table summarizing pTRE-RiniG constructs with variable features. UP region is the region between 5′SS and pPAS, and DN region between 3′SS and pPAS. There are two variants for UP size, DN size, 5′SS type and 3′SS, respectively. There are four variants for the pPAS. Mutants of 5′SS, 3′SS and PAS are highlighted in red. The MaxEnt ranks for 5′SS and 3′SS are indicated. The vertical lines in 5′SS and 3′SS sequences indicate splice site. The vertical line in PAS sequence indicates cleavage site. ‘…’ in PAS sequence indicates sequence not shown. (**D**) Log_2_(red/green) for all pTRE-RiniG constructs transfected in HeLa Tet-On cells. This value indicates relative activities between IPA and splicing. (**E**–**I**). Bar graphs showing Δlog_2_(red/green) indicating isoform changes in cells transfected with target gRNA versus Ctrl gRNA along with various pTRE-RiniG vectors that differed in PAS strength (E), 5′SS strength (F), 3′SS strength (G), UP size (H) and DN size (I). The significance of difference of each value compared to 0 (no change) is indicated (student's *t*-test). n.s. not significant; ***, *P* < 0.01. *P*-values (Wilcoxon test) indicating significance of difference between bars are also shown.

We next wanted to test CRISPRpas for IPA regulation, where CPA is coupled with splicing (illustrated in Figure 5A). To this end, we first constructed a reporter plasmid based on the IPA site of *CSTF3* gene, a conserved site we previously found to be critical for *CSTF3* regulation (40). The construct series was named pTRE-RiniG (RFP intron IRES EGFP), where the IPA site of *CSTF3* (pPAS) was flanked by 5′ and 3′ splice sites (SSs) of the intron 3 of human *CSTF3* gene. This reporter could express two isoforms, an IPA isoform using the pPAS and a splicing isoform using the dPAS (Figure 5B). Because the IPA isoform encodes RFP and the splicing isoform encodes both RFP and EGFP, the red to green fluorescent signal ratio can measure the relative expression levels of the two isoforms (Figure 5B).

To examine the impact of different splicing and PAS features on CRISPRpas regulation of IPA, we altered various features that could impact CPA or splicing (summarized in Figure 5C). IPA site strength changes were based on mutation of upstream AUUAAA hexamer to AAUAAA, or deletion of downstream GUGU element. 5′SS variants were the weak, wild type of intron 3 of *CSTF3* and its mutated, strong version [1st- and 95th-percentiles, respectively, of all 5′SS in the human genome based on maximum entropy (MaxEnt) score (41), Figure 5C], and 3′SS variants were the strong, wild-type of intron 3 of *CSTF3* and its mutated, weak version [94th- and 4th-percentiles, respectively, of all 3′SS in the human genome based on MaxEnt score (41), Figure 5C]. Two additional variants having different upstream distance (UP size, from the 5′SS to the PAS) or downstream distance (DN size, from the 3′SS to the PAS) were also constructed. Flow cytometry analysis indicated variation of IPA isoform versus splicing isoform ratio across these constructs (Figure 5D).

Using one gRNA targeting a region near the 3′SS (Figure 5B), we examined the effectiveness of CRIPSRpas on different variant plasmids. For plasmids with variable PAS strengths (Figure 5E), we found that medium strength PASs (pRiniG-b and pRiniG-f) were more sensitive to CRISPRpas than weak (pRiniG-e) or strong PAS (pRiniG-d) ones (Figure 5E). This might be because a strong PAS could trigger CPA without the involvement of CRISPRpas, whereas a weak PAS does not lead to efficient CPA even with the help of CRISPRpas.

Consistent with the IPA site strength analysis result, strengthening of the 5′SS, which significantly inhibited IPA to the level comparable to that of the weakest PAS (compare constructs g and e, Figure 5D), made CRISPRpas less effective (Figure 5F). In addition, weakening of 3′SS, which significantly activated IPA to the level comparable to that of strongest IPA site (compare constructs h and d, Figure 5D), also made CRISPRpas less effective (Figure 5G). On the other hand, increasing the distance between 5′SS and PAS, which weakens IPA (compare constructs c and b, Figure 5D), made CRISPRpas more effective (Figure 5H). Increasing the distance between 3′SS and PAS, which greatly strengthened IPA (compare constructs a and b, Figure 5D), made CRISPRpas less effective. Together, these data indicate that CRISPRpas regulates IPA and, similar to 3′UTR APA regulation, the baseline level of IPA is important for the effectiveness of CRISPRpas.

### CRISPRpas regulates IPA of endogenous genes

**Figure 6.**
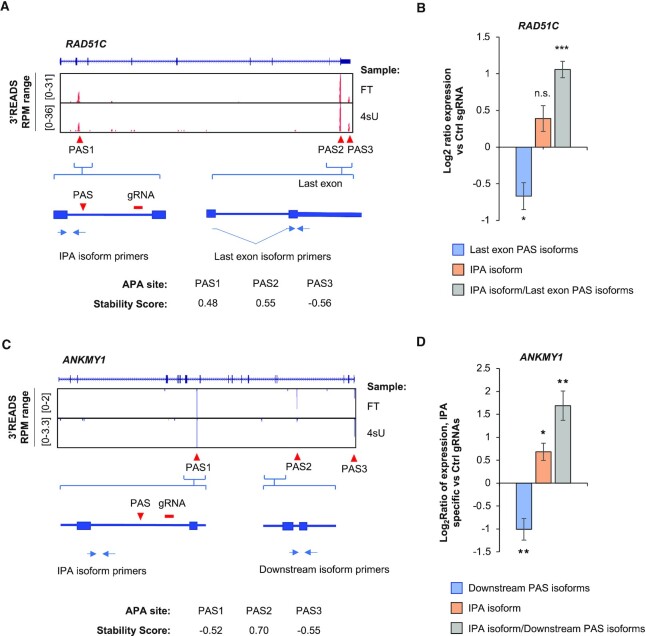
CRISPRpas alters intronic APA of endogenous genes. (**A**) UCSC tracks of human *RAD51C* gene structure and 3′READS+ data for FT and 4sU fractions (top). The three APA sites and Stability Scores are indicated (bottom). A gRNA was designed to target a region in intron 2. Primers for detection of IPA isoform and last exon PAS isoforms are indicated. (**B**) RT-qPCR analysis of relative amounts of last exon PAS isoforms (blue) and IPA isoform (orange) in HEK293T^dCas9^ cells transfected with target or Ctrl gRNAs (48 h). The ratio of IPA isoform/last exon PAS isoform is also shown (gray). Data were normalized to Ctrl gRNA. Error bars are from standard error of mean (*n* = 3). *P-*value is calculated from student's *t*-test. n.s., not significant; *,*P* < 0.05; **, *P* < 0.01; ***, *P* < 0.001. (**C**) As in (A), except that human *ANKMY1* gene structure and data are shown. A gRNA was designed to target a region in intron 7. (**D**) As in (B), except that data for *ANKMY1* are shown. Error bars are standard error of means (*n* = 4).

We next tested CRISPRpas on IPA of endogenous genes. The gene *RAD51C* is known for its function in DNA repair and its mutations have been implicated in various cancers (42,43). Using our HEK293T 3′READS+ data, we identified one IPA isoform using a PAS in intron 2 and two last exon APA isoforms (Figure 6A). Notably, the IPA isoform was previously found to be regulated by termination factors (44). We designed a gRNA targeting a region in intron 2 that was 2260 nt downstream of the IPA site, and transfected it into HEK293T^dCas9^ cells (Figure 6A). Using RT-qPCR with primers specific for the IPA isoform or last exon isoforms (illustrated in Figure 6A), we found that CRISPRpas increased IPA isoform expression and decreased last exon isoform expression (Figure 6B), supporting its effectiveness.

We also tested CRISPRpas on *ANKMY1*, for which we found in our 3′READS+ data one IPA isoform using a PAS in intron 7 as well several other downstream IPA and last exon APA isoforms (Figure 6C). Notably, a human SNP (rs13394744) was found to change the AAUAAA motif to AAUUAA for the IPA site in intron 7 (45). We designed a gRNA targeting a site that was 2099 nt downstream of the IPA site in intron 7 (Figure 6C). RT-qPCR analysis with a primer pair specific for the IPA isoform and another pair for downstream isoforms (Figure 6C) indicated that CRISPRpas increased IPA isoform expression and decreased downstream isoform expression (Figure 6D). Taken together, our data indicate that CRISPRpas works well in promoting IPA of endogenous genes.

### Activation of IPA of *PCF11* by CRISPRpas

**Figure 7.**
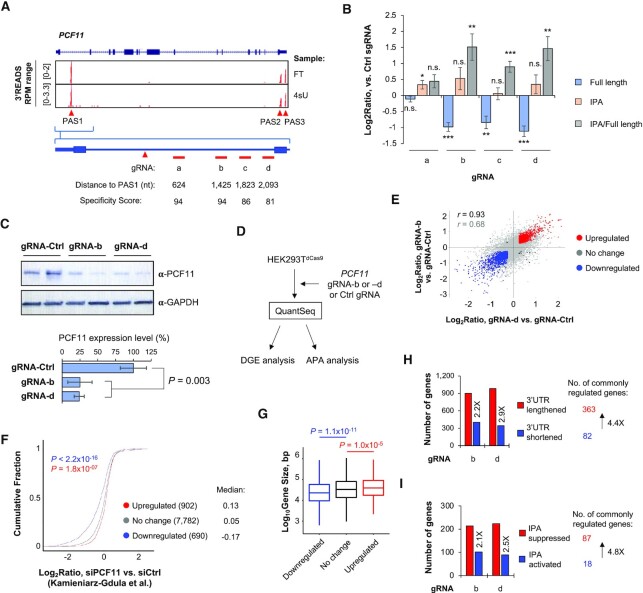
CRISPRpas regulates PCF11 expression through IPA. (**A**) UCSC tracks showing human *PCF11* gene structure (top) and 3′READS+ data for FT and 4sU samples (middle). Intronic PAS (PAS1) and last exon PASs (PAS2 and PAS3) are indicated. Schematic showing gRNA target sites in intron 1 (a–d) are indicated at bottom. Their distances to PAS1 are also shown. (**B**) RT-qPCR analysis of relative amounts of full length (blue) and IPA isoforms (orange) in HEK293T^dCas9^ cells transfected with indicated gRNAs (24 h). Chemically synthesized gRNAs were used. Log_2_(ratio) of expression was normalized to Ctrl gRNA. The ratio of IPA to full length isoforms is also shown (gray). Error bars are standard error of mean (*n* = 3). (**C**) Western blot analysis of PCF11 protein expression in HEK293T^dCas9^ cells transfected with gRNA-b and gRNA-d (72 h after transfection). Expression was normalized to GAPDH. Quantification is shown at the bottom. Error bars are standard deviation (*n* = 2). *P-*value (t-test) is shown to indicate significance of difference in PCF11 expression between samples. (**D**) Schematic showing HEK293T^dCas9^ cells transfected with gRNA-b, gRNA-d, or Ctrl gRNA, followed by RNA sequencing by using the QuantSeq method. Data were subject to differential gene expression (DGE) analysis and APA analysis. (**E**) Scatterplot showing gene expression changes by gRNA-b (y-axis) versus by gRNA-d (x-axis). Significantly upregulated genes in both samples are shown in red, and those downregulated in both samples are in blue. Genes that are significantly regulated in only one sample are shown in black. Other genes are in gray. Pearson correlation coefficients are indicated for all genes (gray) and for commonly regulated genes (black). (**F**) Log_2_(ratio) of three gene groups in RNA-seq data from the Kamieniarz-Gdula *et al.* study, in which *PCF11* was knocked down in HeLa cells. The three gene groups are red, blue and gray genes defined in (E). *P*-values (K-S test) are based on comparison of upregulated (red) or downregulated (blue) genes with no change (gray) genes. (**G**) Box plots of gene size (bp, log_10_) in three gene groups as defined in (E). *P*-values (K-S test) are based comparison of upregulated (red) or downregulated (blue) genes with no change genes. (**H**) 3′UTR APA analysis using MAAPER. Two QuantSeq samples were analyzed individually. The number of genes showing lengthened 3′UTRs (red) or shortened 3′UTRs (blue) are indicated. Commonly regulated genes between two samples are shown, which indicates a global trend of 3′UTR lengthening (genes showing lengthening outnumber those showing shortening by 4.4-fold). (**I**) As in (H), except that IPA analysis result using MAAPER is shown. The number of genes showing suppressed IPA (red) or activated IPA (blue) are indicated. This result indicates global IPA suppression (genes showing IPA suppression outnumber those showing activation by 4.8-fold).

We and others previously identified a conserved IPA site in human and mouse *PCF11* genes (28,46). The IPA site is involved in autoregulation of *PCF11* expression (28,46). Our 3′READS+ data showed three prominent APA sites of *PCF11* used in HEK293T cells, including the IPA site in intron 1 and two PASs in the last exon (Figure 7A).

We designed four synthetic gRNAs targeting the downstream region of IPA site in intron 1, gRNAs-a to -d (Figure 7A) with distances to the IPA site being 624, 1425, 1823 and 2093 nt, respectively (Figure 7A). We transfected these gRNAs into HEK293T^dCas9^ cells, and examined IPA isoform versus full length (FL) isoform (using PASs in the last exon) levels by isoform-specific primer pairs. We found that, except for gRNA-a, all sgRNAs significantly decreased FL isoform expression and increased IPA/FL isoform ratio after 48 h of transfection (Figure 7B). The ineffectiveness of gRNA-a might be attributable to its close distance to the IPA site, despite its high specificity score (Figure 7A).

Using western blotting, we found that gRNA-b and gRNA-d both downregulated *PCF11* full length protein by 75% after 72 h of transfection (Figure 7C), in line with the notion that activation of IPA inhibits PCF11 expression (28,46).

To further explore the consequences of activation of *PCF11* IPA by CRISPRpas, we subjected total RNAs from cells transfected with gRNA-b and gRNA-d to RNA sequencing using the QuantSeq FWD method (Figure 7D, and see ‘Materials and Methods’ section for detail). Comparison of gene expression changes by gRNA-b versus those by gRNA-d showed that these gRNAs regulated a similar set of genes, indicating low off-target effects (*r* = 0.93 and = 0.68 for commonly regulated and all genes, respectively, Pearson correlation, Figure 7E).

We next identified genes commonly upregulated or downregulated in gRNA-b and gRNA-d samples, and examined their gene expression changes in the data previously generated by Kamieniarz-Gdula *et al.*, which corresponded to knocking down of PCF11 using siRNAs in HeLa cells (46). We found that genes downregulated by gRNA-b and gRNA-d were also significantly downregulated in the Kamieniarz-Gdula *et al.* data (*P* < 2.2 × 10^−16^, K–S test, Figure 7F) compared to genes without expression changes by gRNA-b and gRNA-d treatments. Conversely, the genes upregulated by gRNA-b and gRNA-d were also significantly upregulated in the Kamieniarz-Gdula *et al.* data (*P* = 1.8 × 10^−7^, K–S test, Figure 7F). Consistent with the notion that PCF11 regulates gene expression based on gene size (46,47), we found that genes downregulated by gRNA-b and gRNA-d were significantly smaller than non-regulated genes (*P* = 1.1 × 10^−11^, Wilcoxon test, Figure 7G), and those upregulated by gRNA-b and gRNA-d were significantly larger than non-regulated genes (*P* = 1.0 × 10^−5^, Wilcoxon test, Figure 7G).

Because PCF11 globally regulates APA (46,47), we applied the MAAPER program to examine APA using our QuantSeq data. We found that CRISPRpas with gRNA-b or gRNA-d led to more genes with 3′UTR lengthening than with 3′UTR shortening (Figure 7H). For commonly regulated events, genes with 3′UTR lengthening outnumbered those with 3′UTR shortening by 4.4-fold (363 versus 82).

We also analyzed IPA regulation using our QuantSeq data and the MAAPER program. Consistent with PCF11′s function, CRISPRpas with gRNA-b or gRNA-d led to more genes with IPA suppression than those with IPA activation (Figure 7I). For commonly regulated events, genes with IPA suppression outnumbered those with IPA activation by 4.8-fold (87 versus 18). Taken together, our data indicate that CRISPRpas effectively promotes IPA of *PCF11*, leading to downregulation of its protein and hence widespread changes in gene expression and APA.

## DISCUSSION

In this study, we report a novel CRISPR/dCas9-mediated method to regulate gene expression through APA. We show that CRISPRpas induces mRNA isoform abundance changes for genes harboring APA sites in 3′ UTRs or introns. Using reporter constructs, we found that PAS strength and the distance between the PAS and target site are critical factors for the efficacy of CRISPRpas. In addition, we demonstrate the utility of CRISPRpas in APA regulation of endogenous genes. Moreover, by detailed analysis of *PCF11*, we show activation of its IPA leads to downregulation of protein expression, resulting in widespread APA and gene expression changes.

We found that PAS strength is an important feature for CRISPRpas-mediated regulation. When a PAS is very weak, such as the BD version of *CSTF3* IPA site (Figure 2), CRISPRpas could lead to pre-mRNA degradation, similar to the effect of CRISPRi (25). When PAS strength is at medium level or higher, such as the AD and AE versions (Figure 2), CRISPRpas leads to CPA at the PAS. The distance between PAS and CRISPRpas target site is presumably important for recognition of the PAS by the CPA machinery. As such, the greater the distance between target site and the PAS, the better the effect of CRISPRpas. This notion is also consistent with our results on CRISPRpas of endogenous genes.

When a PAS is located in an intron, the PAS usage at the base line is important for the efficiency of CRISPRpas. IPA activity is under the control of both splicing and CPA (20). Therefore, when IPA activity is very low, e.g. in introns with strong 5′SS and/or 3′SS, or when IPA activity is very high, e.g. in introns with weak 5′SS and/or 3′SS, the effect of CRISPRpas is mitigated. In those cases, increasing the distance between PAS and target site and using multiple target gRNAs might be advisable.

CRISPRpas offers several advantages over other previously used methods for regulation of APA. Conventional Cas9-mediated gene editing of PAS has been used to manipulate APA of a gene, for example, addition of PAS to the CDS end of *CCND1* gene (27) and deletion of *PCF11* intronic PAS (28,46). However, genome editing by Cas9 requires extensive manipulation of the cell, which could lead to secondary or indirect effects. By contrast, CRISPRpas offers a programmable platform to regulate APA in a short time frame. If coupled with inducible expression of dCas9 and/or gRNAs, analysis of the consequences of APA can be restricted to a short time window, reducing secondary effects.

## DATA AVAILABILITY

Sequencing datasets generated in this study have been deposited into the GEO database under the accession number GSE161727.

## Supplementary Material

gkab519_Supplemental_Files
