# Supplementary material for: CRISPRpas: programmable regulation of alternative polyadenylation by dCas9
Source: Nucleic Acids Res. 2021 Jul 9;50(5):e25. doi: 10.1093/nar/gkab519 (PMC8934653; doi:10.1093/nar/gkab519)
Supplement: gkab519_Supplemental_Files [file gkab519_supplemental_files.zip › CRISPRpas-Supplementary Data.pdf]

Supplementary Data

CRISPRpas: programmable regulation of alternative polyadenylation by dCas9.

Shin et al.

Supplementary Table S1. Plasmids used in this study.

Plasmid name	Construction detail
pRiG	The AD and AE variants were from Ji et al., 2009. BD variant was constructed by cloning a fragment containing part of the intronic polyA site of *CSTF3* into pCMV-RiG (Pan et al., 2006) by using PCR (5'-CGATGAATTCTGATGATTGTTGCATTTT-3' and 5'-GGCCGAATTCACAAGTAAATAAAAGGCT-3') and restriction enzyme EcoRI.
pTRE-RiG-TIMP2	The proximal PAS of TIMP2 was amplified from HEK293T genomic DNA. Both PCR product and pTRE-RiG-AD were digested with XhoI and SalI, followed by ligation.
pGR9	The Cas9 gRNA scaffold from pX459 (Addgene #62988) was subcloned into pXR003 (Addgene #109053) by using NdeI and EcoRI sites.
CMV-dCas9-FKBP	The dCas13 sequence of pAC1807 (Addgene #119740) was swapped with the dCas9 sequence from pHR-SFFV-dCas9-BFP-KRAB (Addgene #46911) by using AscI and BamHI sites.
PB-CAG-dCas9-10xGCN4-P2A-BFP	The dCas9-10xGCN4-P2A-BFP sequence from pHRdSV40-dCas9-10xGCN4-P2A-BFP (Addgene #60903) was subcloned into PB-CAG-BGHpA (Addgene #92161) by using MluI and NheI sites.
pTRE-RiniG	The intronic region of pRinG vector (Luo et al., 2013) was inserted into pTRE-RiG-TIMP2 by using XhoI and BamHI sites, creating pTRE-RiniG. Other intronic variants (Luo et al., 2013) were subcloned into this vector by using XhoI and SalI sites.

Supplementary Table S2. Oligos used for gRNA cloning.

Target gene or region	gRNA name	Sense sequence	Antisense sequence
Ctrl	a	CACCTTCTCTTGCTGAAAGCTCGA	AAACTCGAGCTTTCAGCAAGAGAA
Ctrl	b	CACCGTTAGACACGAACACCACGGT	AAACACCGTGGTGTTCGTGTCTAAC
Ctrl	c	CACCGCAGGTCGTACTTGTCGATCA	AAACTGATCGACAAGTACGACCTGC
Ctrl	d	CACCGTGTTCCAGCACGACGACGG	AAACCCGTCGTCGTGCTGGAACAC
pTRE-RiG-TIMP2	a	CACCGGACCAGTCGAAACCCTTGG	AAACCCAAGGGTTTCGACTGGTCC
pTRE-RiG-TIMP2	b	CACCGCCAGGAAGGGATGTCAGAGC	AAACGCTCTGACATCCCTTCCTGGC
pTRE-RiG-TIMP2	c	CACCGAATAAAACACTCATCCCAT	AAACATGGGATGAGTGTTTTATTC
pTRE-RiG-TIMP2	c	CACCGACCCACAACCATGTCTAAA	AAACTTTAGACATGGTTGTGGGTC
pTRE-RiG-TIMP2	e	CACCGTTTAGACATGGTTGTGGGTC	AAACGACCCACAACCATGTCTAAAC
IRES	NT	CACCGCTTCGGCCAGTAACGTTAG	AAACCTAACGTTACTGGCCGAAGC
IRES	T	CACCGCGTTACTGGCCGAAGCCGCT	AAACAGCGGCTTCGGCCAGTAACGC
EGFP	NT1	CACCGACCAGGATGGGCACCACCC	AAACGGGTGGTGCCCATCCTGGTC
EGFP	NT2	CACCGCCGTCCAGCTCGACCAGGAT	AAACATCCTGGTCGAGCTGGACGGC
EGFP	NT3	CACCGGTGGTGCAGATGAACTTCA	AAACTGAAGTTCATCTGCACCACC
EGFP	NT4	CACCGCACGGGGCCGTCGCCGATGG	AAACCCATCGGCGACGGCCCCGTGC
EGFP	T1	CACCGGGCGAGGAGCTGTTCACCG	AAACCGGTGAACAGCTCCTCGCCC
EGFP	T2	CACCGGTGCCCATCCTGGTCGAGC	AAACGCTCGACCAGGATGGGCACC
EGFP	T3	CACCGGCCACAAGTTCAGCGTGTC	AAACGACACGCTGAACTTGTGGCC
EGFP	T4	CACCGTGAACCGCATCGAGCTGAA	AAACTTCAGCTCGATGCGGTTCAC
EIF1AD	a	CACCGAGGTGAGCACTCCCGTACAC	AAACGTGTACGGGAGTGCTCACCTC
EIF1AD	b	CACCGTAAACGTAAGGTACAACTGG	AAACCCAGTTGTACCTTACGTTTAC
EIF1AD	c	CACCGAAGTCCCATTGGCTTACGAC	AAACGTCGTAAGCCAATGGGACTTC
TIMP2	a	CACCGAAAGCCCCGTGCAGAACGA	AAACTCGTTCTGCACGGGGCTTTC
TIMP2	b	CACCGTACAGCATGAAAACGCCCGT	AAACACGGGCGTTTTCATGCTGTAC
CCND1		CACCGACTCTGGGAAACGCCAAAC	AAACGTTTGGCGTTTCCCAGAGTC
CKS1B		CACCGAAAACCTTCCCCAACCAAG	AAACCTTGGTTGGGGAAGGTTTTC
pTRE-RiniG-800	intronic	CACCGGCTACCACTGTGACAAGAC	AAACGTCTTGTCACAGTGGTAGCC
RAD51C		CACCGTAAATCAACTGGATTCGGGG	AAACCCCCGAATCCAGTTGATTTAC

Supplementary Table S3. Synthetic gRNAs used in this study.

**Target gene or region**	**sgRNA name**	**Target sequence (without PAM)**
Ctrl	a	TTCTCTTGCTGAAAGCTCGA
EGFP	NT2	CCGTCCAGCTCGACCAGGAT
TIMP2	a	GAAAGCCCCGTGCAGAACGA
PCF11	a	ATTTAGTTTTCGGTTCTCGA
PCF11	b	TTTACCCACCCCACGTGTAT
PCF11	c	GCCGCTGAATCATGTAAATC
PCF11	d	TGACCCATATTAACTTACAA

Supplementary Table S4. RT-qPCR primers used in this study.

Gene	Region	Forward sequence	Reverse sequence
EIF1AD	cUTR	aggacccaattctccacttgc	tcatgcaggggtgaagatgtg
EIF1AD	aUTR	tgagtattgtgtctgggtgtgg	acgcctgtttcagaaatggg
TIMP2	cUTR	ccatgatcccgtgctacatct	gtcgagaaactcctgcttgg
TIMP2	aUTR	ctaagcacagctctcttctcct	cagcataaacacagtgctccc
CCND1	cUTR	ttgctctttccccсttccatc	ccgctcagggttatgcaaatac
CCND1	aUTR	acgcttacctcaaccatcctg	acaacatccaggacttgtgc
CKS1B	cUTR	agctacttttcagcctcaagc	agcaaagcagttaccagcac
CSK1B	aUTR	atagcaggcatcaacatcgg	aacattgctgtgcccaatcc
PCF11	IPA	tatccagagcggcttcagcttc	cggggtgtataaaaaccttgg
PCF11	FL	aaggcacagttggcagtttc	cggcttttctcagattgcac
RAD51C	IPA	actggaacttcttgagcaggag	tgcacatacacacttaaaattaagagcaca
RAD51C	FL	actgtttcaaatcaaacctcagg	aattcttcctctgggtctcgtg
ANKMY1	IPA	tccatgatcgagtaggtcctg	agcataggattcagccctgatg
ANKMY1	FL	tcctgaagcctgtaatgctcag	tccggtcctggaagaatctg
GAPDH	-	tcaccaccatggagaaggc	gctaagcagttggtggtgca

cUTR, common 3’UTR; aUTR, alternative 3’UTR; IPA, IPA isoform; FL, full length isoform.

Supplementary Table S5. Stability Scores of all poly(A)+ transcripts detected by 3'READS in HEK293T cells. PAS ID is PAS identification comprising chromosome number, genomic position and genomic strand. Chr, chromosome number; Pos, genomic position; Str, genomic strand; Location, PAS location in the gene, i.e., intron, 3'UTR, or not assigned (NA). Gene Symbol, NCBI Gene Symbol of the gene to which PAS is assigned. When a PAS is not assigned to a gene, the Gene Symbol is NA. Stability Score (SS) of replicate 1 and replicate 2 are SS Rep1 and SS Rep2, respectively. A poly(A)+ transcript is considered detected if there are at least five reads in both replicates.

Figure S1

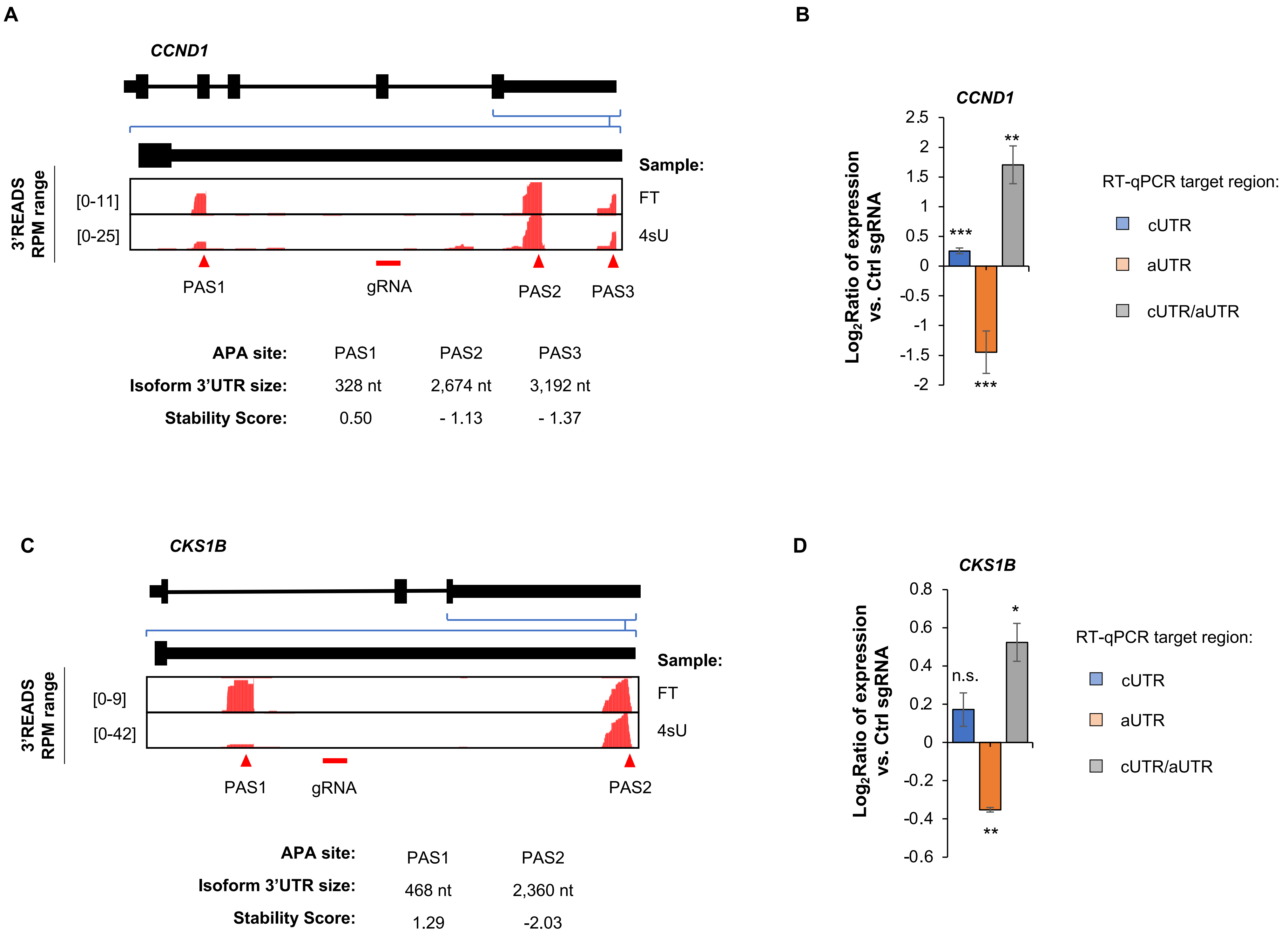

Figure S1. Two additional genes whose 3'UTR APA is regulated by CRISPRpas. As in Figure 4, except that *CCND1* **(A)** and *CKS1B* **(B)** data are shown. Error bars are standard error of mean (n = 3 and 2, respectively).

Figure S2

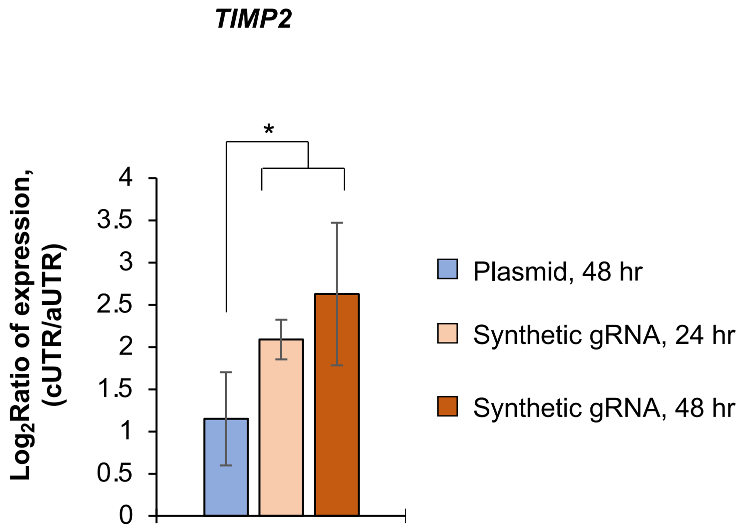

Figure S2. Comparison of plasmid-encoded gRNAs with synthetic gRNAs for CRIPSRpas. Log_2(Ratio) of *TIMP2* cUTR expression vs. aUTR expression was analyzed by RT-qPCR with RNA from HEK293T^{dCas9} cells transfected with a plasmid-encoded *TIMP2* gRNA (gRNA-a) or a chemically synthesized gRNA corresponding to gRNA-a. Cell harvest time is indicated. Error bars are standard error of mean (n = 6, 2, and 2, respectively). *P*-value (Student's t-test) for significance of difference between samples is indicated. *, P <0.05.
